# Chronic Inflammatory Demyelinating Polyneuropathy and Concurrent Membranous Nephropathy Associated With Anti-Contactin-1 Autoantibodies: A Rare Case Report With a Review of the Literature

**DOI:** 10.7759/cureus.83975

**Published:** 2025-05-12

**Authors:** Ola Tarabzuni

**Affiliations:** 1 Department of Nephrology, King Abdulaziz University Hospital, Jeddah, SAU

**Keywords:** anti-contactin-1 antibodies, case, chronic inflammatory demyelinating polyneuropathy (cidp), membranous nephropathy, nephropathy

## Abstract

Membranous nephropathy (MN) stands as the most common origin of nephrotic syndrome in adults. Nevertheless, it is quite unusual for individuals to simultaneously manifest both chronic inflammatory demyelinating polyneuropathy (CIDP) and MN along with the presence of positive anti-contactin-1 (CNTN1) antibodies. Only a limited number of case reports in scientific literature have described such occurrences to date. Typically, CIDP patients exhibit symptoms characterized by proximal and distal weakness and sensory abnormalities. We present a rare case of CIDP and MN with positive anti-CNTN1 antibodies in our setting and describe our experience in management of the condition. Moreover, noticing the rarity of this condition, we performed an analysis of the existing literature to comprehensively analyze the diagnostic, management, and clinical outcomes for this condition. Our patient, a 45-year-old male, had a pre-existing diagnosis of CIDP at the age of 43, in March 2015. Approximately 18 months later, in September 2016, this patient presented with nephrotic syndrome, leading to a subsequent diagnosis of stage 2 MN. The diagnosis was confirmed through renal biopsy results, which revealed thickening of the glomerular basement membrane and immunoglobulin G4 (IgG4) deposits. However, the patient tested negative for anti-phospholipase 2 antibody. Further diagnostic evaluation was performed, and anti-CNTN1 antibodies were detected. The patient was successfully treated with cyclosporine therapy 150 mg twice a day and prednisone, and no complications were noted; however, partial relapse on remission of cyclosporine was observed. Based on our case analysis and comprehensive review of existing literature, it is evident that there are similarities between CIDP with MN and positive anti-CNTN1 antibodies, but they are not identical conditions. Therefore, we propose the assessment of anti-CNTN1 antibodies as part of the evaluation for patients who exhibit both CIDP and MN symptoms. Anti-CNTN 1 antibody may be a novel diagnostic test in this condition and may allow determination of therapeutic response soon; however, this needs to be backed up with evidence from research studies in the future.

## Introduction

Membranous nephropathy (MN) is a rare disease that causes the immune system to attack the glomeruli, which are the filters in the kidneys [1]. This affects the podocytes and results from the development of immunological deposits on the subepithelial side of the glomerular capillary wall, which results in its inflammation. Immunoglobulin G, which is directed against antigens that have long been unknown, comprises these immunological deposits. When MN occurs in combination with other clinical diseases such as autoimmune diseases, cancers, infections, and hepatitis B, it is referred to as secondary MN and accounts for almost 30% of cases while in the majority 70% of cases, MN is not associated with any recognized disease and is classified as primary MN [2]. MN may affect anyone at any stage of life. It is the most common cause of nephrotic syndrome in adults. The thickening of the glomerular capillary walls caused by immune complex deposition is a characteristic of MN, an autoimmune disease. It is characterized by massive proteinuria (>3.5 g/day) and clinically presents with peripheral edema, hypertension, frothy urine, and manifestations of thromboembolic phenomena [1]. Since the phospholipase A2 receptor (PLA2R), which is on the surface of podocytes in kidneys, was identified in 2009 as the primary antigen in adults, disease detection and surveillance have undergone a paradigm shift, with the characterization of numerous other antigens. PLA2R is clinically important as the major autoantigen in MN, a kidney disease characterized by subepithelial immune complex deposition leading to nephrotic syndrome. In most cases of MN, circulating anti-PLA2R autoantibodies can be detected in the serum, and PLA2R antigen can be found in immune deposits in kidney biopsy samples. The presence of anti-PLA2R antibodies is used for diagnosis, prognosis, and monitoring treatment response in MN [3]. However, it is challenging to forecast the course of the disease, and about one-third of individuals will experience spontaneous remission [4].

Simultaneous occurrence of MN and chronic inflammatory demyelinating polyradiculoneuropathy (CIDP) is quite a rare phenomenon. Witte and Burke initially described the association between MN and CIDP in 1987. Since then, few and limited concurrent cases have been described in the literature, highlighting the rare co-occurrence of these conditions [5]. CIDP is an immune-mediated demyelinating neuropathy characterized by symmetric weakness in proximal and distal muscles and sensory abnormalities that progress gradually or in a recurring manner over a span of approximately two months. While both humoral and cell-mediated immunity are believed to play a role in CIDP, its exact underlying cause remains unidentified. Nevertheless, recent research has shed light on the presence of autoimmune antibodies in specific subsets of CIDP patients, targeting nodal-paranodal cell-adhesion molecules such as neurofascin-155, contactin-1, contactin-associated protein 1, and various neurofascin isoforms. In accordance with the most recent guidelines, individuals with autoantibodies against paranodal proteins are categorized as having autoimmune nodopathies, as they often exhibit distinctive clinical features and pathological manifestations [6].

The co-occurrence of CIDP and MN, a primary etiology of adult nephrotic syndrome, has been documented in some case reports [6-12]. A case series reported six patients with both CIDP and MN, of whom four were positive for anti-CNTN1 antibodies. However, due to the limited number of reported cases, the exact prevalence of this association remains unclear. The identification of autoantibodies against podocyte antigens, specifically PLA2R and thrombospondin type 1 domain containing 7A, convincingly defined the autoimmune character of idiopathic MN. Positive anti-contactin-1 (CNTN1) antibody is rarely identified. CNTN1 is an adhesion molecule that is a member of the immunoglobulin superfamily. Together with glial NF155, axonal CNTN1 and CASPR1 establish septate-like junctions that sustain ion channel clustering at Ranvier nodes [7,13]. Since CIDP is an uncommon condition, fewer than 10% of patients have IgG4 anti-CNTN1 antibodies. As a result, there are few documented cases of concurrent MN and anti-CNTN1 antibody-positive CIDP, which limits the understanding of the relationship between the two autoimmune disorders. Anti-CNTN1 antibodies have a well-established role in CIDP, as evidenced by their disruption of axo-glial contacts at the paranodes; however, their function in MN is still unknown. The majority of individuals with concurrent MN and CIDP have either appeared with both conditions at the same time or within two months of each other's diagnosis, which may point to a shared antigenic target. Although the function of anti-CNTN1 autoantibodies in the conjunction of CIDP and MN is unknown however, in patients with aggressive CIDP who also have positive CNTN1 autoantibodies, the presence of CNTN1 antibodies may be a unique biomarker and prompt further evaluation [8].

In this case report we describe a case of CIDP and MN with positive anti-CNTN1 antibodies. Through our case we hypothesize the contributory role of CNTN1 antigen in co-occurrence of CIDP and MN. We will also describe how the presence of presence of anti-CNTN1 antibodies contribute to the pathogenesis of both conditions, as well as the differences from other reports in terms of clinical presentation or response to treatment. Additionally, keeping in mind the rarity of this occurrence, we performed analysis of the existing literature in the PubMed database for the past 10 years. Our search results included a total of seven studies with six case reports and one case series comprising a total of 14 cases among which anti-CNTN1 antibodies were detected in eight cases only while one study did not report regarding anti-CNTN1 antibodies [6-12].

## Case presentation

**Figure 1:**
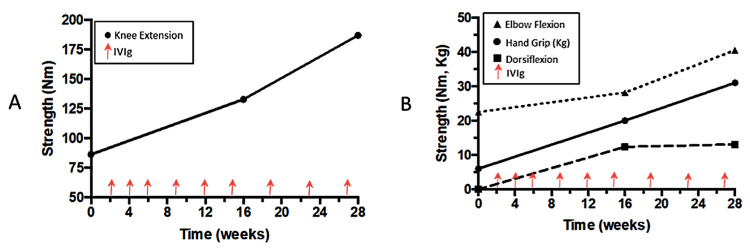
EMG Electromyograph (A) Strength increased significantly with intravenous immunoglobulin therapy over 28 weeks of therapy for quadriceps. (B) Hand grip, elbow flexors and dorsiflexors. Median and peroneal motor conduction parameters uniformly improved over 105 weeks of therapy. Nm: newton-metre, Kg: kilogram, IVIg: Intravenous immunoglobulin

A 45-year-old male with no prior significant medical, surgical, or family history was diagnosed with CIDP in March 2015. Treatment was initiated with prednisone, intravenous immunoglobulin (IVIG), and azathioprine, resulting in notable clinical and electrophysiologic improvements. Specifically, strength gains were observed in the quadriceps (Figure 1A), hand grip, elbow flexors, and dorsiflexors (Figure 1B) during the first 28 weeks of IVIG therapy. Median and peroneal motor conduction parameters also showed consistent improvement over a 105-week treatment period.

In March 2016, the patient developed symptoms of pulmonary embolism and was treated successfully with rivaroxaban.

Six months later, in September 2016, he presented with features of nephrotic syndrome. Urinalysis revealed 22 g/day of proteinuria. Additional findings included anasarca, hypoalbuminemia, and hypercholesterolemia. Renal function remained normal, and tests for anti-phospholipase A2 receptor antibodies, malignancy, infections, and routine autoimmune markers were all negative.

**Figure 2:**
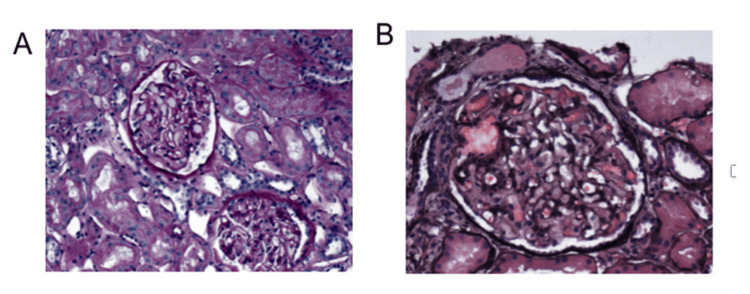
(A) PAS stain showed mild thickening of glomerular basement membrane. (B) Jones silver shows essentially normal appearing glomerular basement membrane. PAS: Periodic acid-Schiff

Renal biopsy was performed. Light microscopy showed mild thickening of the glomerular basement membrane with Periodic acid-Schiff (PAS) staining (Figure 2A) and essentially normal appearance with silver staining (Figure 2B).

**Figure 3:**
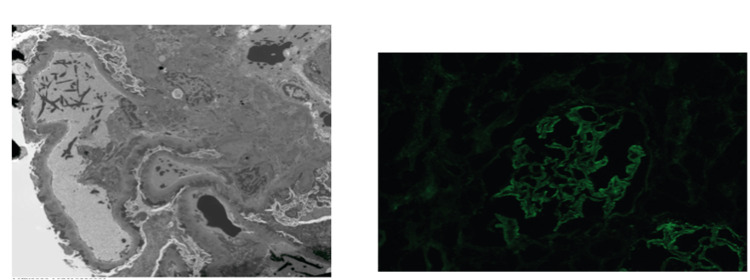
(A) Electron microscopy shows glomerular basement membrane and interstitial dense deposits suggestive of immune globulin. This was diagnostic of stage 2 membranous nephropathy and suggestive of secondary MN. (B) Immunofluorescence shows positive staining for IgG. MN: Membranous nephropathy, IgG: Immunoglobulin G

Electron microscopy revealed glomerular basement membrane and interstitial dense deposits suggestive of immune globulins (Figure 3A), consistent with stage 2 MN, likely secondary. Immunofluorescence was positive for IgG (Figure 3B). 

**Figure 4:**
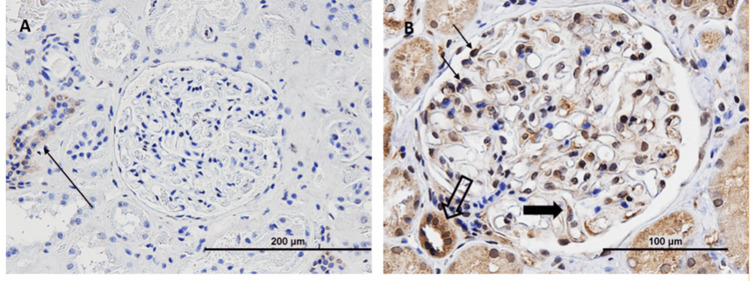
Control (A) and patient sample (B ) A (Control): Shows no contactin-1 staining in the glomerulus, indicating that normal renal tissue does not express or bind contactin-1 in detectable amounts with this method. This rules out background staining or non-specific binding. B (Patient sample): Shows extensive contactin-1 staining in the glomerulus, indicating that contactin-1 antigen is present and likely bound by circulating autoantibodies (IgG4 anti-contactin-1) in the patient’s tissue.

Serum analysis identified circulating IgG4 anti-contactin 1 antibodies, while neurofascin antibodies were negative. Immunohistochemistry of renal tissue demonstrated strong contactin-1 staining in the patient’s glomeruli (Figure 4B), whereas control samples showed no staining (Figure 4A), confirming the presence and binding of contactin-1 antigen by circulating autoantibodies in affected tissue.

The patient was started on cyclosporine 150 mg twice daily along with prednisone. Over 18 months, 24-hour urinary protein decreased from 22 g/day to 0.95 g/day. A partial relapse occurred when cyclosporine was discontinued.

## Discussion

Our case represents a rare instance of the simultaneous presence of CIDP and MN along with positive anti-CNTN1 antibodies. Anti-CNTN1 antibodies differ from PLA2R and THSD7A in MN in origin, frequency, and associated systemic disease. Anti-CNTN1 antibodies signal a broader autoimmune process affecting both nerves and kidneys, unlike PLA2R and THSD7A which are kidney-specific markers for primary MN.

It is noteworthy that despite the absence of anti-PLA2R antibodies, the detection of IgG4 deposition on the kidney's glomerular basement membrane indicates immune-mediated damage to podocytes. Consequently, we assume and predict the role of anti-CNTN1 antibodies in contributing to this condition in our patient. Nephrotic syndrome in adults is primarily caused by MN. There have been several reports of atypical MN occurring concurrently with CIDP. Although the relationship and pathophysiology between anti-CNTN1 antibodies and CIPD/MN are not understood very well, the presence of anti-CNTN1 antibodies contributes to the pathogenesis of both CIDP and membranous nephropathy through an autoimmune mechanism targeting CNTN1, a neuronal and glomerular protein. In CIDP, anti-CNTN1 antibodies-primarily of the IgG4 subclass-bind to CNTN1 located at the paranodal regions of peripheral nerves. This disrupts the axo-glial interactions necessary for maintaining the integrity of the nodes of Ranvier, leading to paranodal demyelination, conduction block, and progressive neuropathy [14]. In membranous nephropathy, anti-CNTN1 antibodies may cross-react with CNTN1 expressed in podocytes, contributing to immune complex deposition and complement activation along the glomerular basement membrane. This leads to podocyte injury, proteinuria, and nephrotic syndrome [15]. The shared target of CNTN1 explains the co-occurrence of neurological and renal manifestations in affected patients. The antibodies mediate pathogenic effects through disrupting adhesion molecules critical for nerve conduction and glomerular filtration barrier integrity [6]. A small subset of individuals with CIDP develop antibodies against various neurofascin and contactin subtypes. Reports of cases of concomitant CIDP and nephrotic syndrome suggested that autoantibodies against myelin and podocytes may be the mechanism generating neuro-renal disease. To our knowledge, there have been 27 cases of CIDP with MN reported in the literature; four of these patients experienced both CIDP and MN symptoms simultaneously, 16 of them experienced CIDP symptoms before MN, and two of them experienced MN clinical manifestations before CIDP nervous system symptoms. Anti-CNTN1 antibodies were identified in five of the individuals [16]. In our case MN developed successively after a year and a half of CIDP, for which he was diagnosed in the year 2015.

Similar to our case, Nazarali et al. also presented a case of a 43-year-old male who developed MN almost a year after CIDP. Renal biopsy was suggestive of stage 2 MN; additionally, the biopsy revealed excessive CNTN1 staining within the glomerulus in comparison to controls [8]. Excessive CNTN1 staining within the glomerulus was also observed in the histopathological analysis of our patient. Additionally, similar to our assumption, Le Quintrec et al. also hypothesized that for MN patients suffering from CIDP, contactin 1 represents a unique shared antigenic target as the authors investigated kidney biopsy specimens from five patients who had both MN and CIDP and were positive for anti-CNTN1 antibodies. Neurological tissue and eluted IgG from biopsy sections were tested against contactin 1. Normal renal glomeruli expressed contactin 1, as shown by a Western blot. Contactin 1 and IgG4 were detected and colocalized on the glomerular basement membrane of these patients by confocal microscopy examination. In individuals with anti-PLA2R1-associated MN or membrane lupus nephritis, or in a healthy control group, glomerular contactin 1 was lacking. The predominant eluted subclass IgG4 was linked to contactin 1 by the eluted IgG from biopsy sections that were positive for contactin 1, but not by the eluted IgG from individuals who had PLA2R1 MN. Eluted IgG was able to colocalize with commercial anti-CNTN 1 antibody and bind to paranodal tissue, which is a myelinated axon. However, the pathophysiology still remains unknown [17].

Our patient was successfully treated with cyclosporine and prednisone and no serious complications were noted. However, partial relapse on remission of cyclosporine was observed. Findings from literature review also exhibit that patients were effectively managed with prednisone, cyclosporine, rituximab, plasma exchange, cyclophosphamide, and immunoglobulin therapeutic strategies. IVIG is the first-line treatment of CIDP. It works by modulating the immune response that causes demyelination of peripheral nerves. Specifically, IVIG blocks Fc receptors on macrophages, reducing immune-mediated nerve damage. It also neutralizes autoantibodies that target myelin and alters cytokine production and T-cell function. IVIG leads to improvement in muscle strength, sensory symptoms, and functional status in many patients. It is often used for initial treatment and for maintenance therapy in relapsing or treatment-dependent cases [18]. No serious complications were observed except in one case whose condition did not improve and the patient was dependent on daily living. Moreover, the findings of our literature review show that almost all patients presented with weakness and paresthesia along with nephrotic syndrome. Renal biopsies also revealed thickening of the glomerular membrane in each of the cases, however anti-CNTN1 antibodies were detected in only eight cases. Additionally, we observed that most of the cases among our included case studies were above 40 years of age, except one female patient who was 33 years old. The detailed summary of the literature review is illustrated in Table 1.

**Table 1:** Clinical characteristics of the included cases NR: not reported; CNTN1: anti-contactin-1; eGFR: estimated glomerular filtration rate, ANCA: antineutrophil cytoplasmic antibodies; LAC: lupus anticoagulant; ANA: antinuclear antibodies; ENA: extractable nuclear antigens; MN: membranous nephropathy; PLA2R: phospholipase A2 receptor; IgG: immunoglobulin G; CIDP: chronic inflammatory demyelinating polyneuropathy; SLE: systemic lupus erythematosus

Author	Year	Age and Gender of case/cases	Symptoms	Diagnostic evaluation	Treatment	Complications	Anti-CNTN1 antibody
Santoro et al. [9]	2022	73 (female)	Proximal asymmetric lower limb weakness with ataxic gait	Laboratory investigations revealed proteinuria, hypoalbuminemia, and an eGFR of 73 ml/min/1.73 m2 while other autoantibodies (ANCA, DNA, LAC, ANA, ENA, and anti-cardiolipin) were not detected. A kidney biopsy revealed a typical picture of MN. Both the PLA2R and THSD7A antigens were not detected in the kidney tissue, and the anti-PLA2R antibody test result was negative.	The patient was initially treated with prednisone at 50 mg/day, which was gradually tapered to 15 mg/day however no significant improvements were noted therefore the patient was switched to 2 infusions of 1g rituximab, 2 weeks apart which resulted in significant improvement overall	None	Detected
Zhang et al. [10]	2023	67 (male)	The patient was unable to walk due to acute edema, weakness, and a progressive rise in limb numbness.	Investigations showed severe proteinuria however antibodies including anti-neurofascin-155 (NF-155), anti-NF-186, anti-CNTN1, anti-CNTN2, and anti-contactin-associated protein-1 were negative. Immunohistochemistry revealed that PLA2R was not detected also. Renal biopsy showed that the glomerular basement membrane was uniformly and diffusely thickened, exhibiting segmental spike development, extensive subepithelial electron-dense deposits, and diffuse foot process effacement, all of which were consistent with atypical MN accompanied with glomerular hypertrophy and ischemia renal damage.	Intravenous methylprednisolone 40 mg daily for approximately two weeks, followed by oral prednisone, and plasma exchange was performed five times which was further followed with 2–4 weeks of rituximab treatment leading to optimal improvement	None	Not detected
Hashimoto et al. [7]	2018	70 (gender NR)	Progressive weakness and superficial and deep sensory impairment in four extremities for over 6 months, edema in both legs and wheeze in right lower lung	Laboratory investigations revealed nephrotic syndrome while renal biopsy showed thickening of the basement membrane along with glomerular IgG4 deposits and local subepithelial projections consistent with MN. Autoantibody assays detected IgG4 and IgG1 anti-CNTN1 antibodies; however, anti-NF155, anti-PLA2R, and anti-THSD7A antibodies were not detected. Sjögren's syndrome was also identified in the patient on the basis of elevated anti-SSA/Ro antibodies and pertinent clinical features.	Oral prednisolone (1 mg/kg/day with a moderate taper) is administered after three days of methylprednisolone pulse treatment (1000 mg/day). Intravenous immunoglobulin (IVIG) (400 mg/kg/day for 5 days) was administered two months after the initial course of treatment. This led to significant improvement in the condition of the patient	None	Detected
Wong et al. [11]	2015	Case 1: 36 (male)	Progressive weakness paraesthesia (glove and socking distribution), edema on both lower legs	Cerebrospinal fluid analysis showed increased levels of protein while immunofluorescence testing on a renal biopsy sample revealed MN with only IgG deposits, primarily of the IgG4 subclass. No amyloid deposition was seen.	Initial treatment comprised intravenous immunoglobulin and prednisolone however symptoms of weakness worsened with prednisolone, so patient was switched to cyclosporin. Later patient was again switched to prednisolone with tacrolimus and further sessions of plasma exchange. No significant improvements were seen	Patient is dependent for activities of daily life	Not reported
		Case 2: 33 (female)	Progressive weakness in hands and feet and lower legs	Proteinuria also cerebrospinal fluid analysis showed increased protein levels while immunofluorescence analysis after renal biopsy revealed MN with IgG deposits, primarily o f IgG2, 3 and 4 subtypes. No amyloid deposition was seen.	High doses of prednisolone were started leading to improvement in symptoms of weakness however proteinuria persisted for which further cyclophosphamide was added. Patients condition improved significantly.	None	Not reported
Xu et al. [6]	2021	57 (male)	Known case of MN, limb numbness and weakness, on walking aid	Using immunofluorescence microscopy, the capillary walls were found to contain deposits of PLA2R (+++) and granular IgG. Upon ultrastructural examination, the glomerular basement membrane showed non-uniform diffuse thickening, diffuse foot process effacement, and numerous deposits of electron-dense material beneath the epithelium and inside the basement membrane, with some of the electron-dense material embedded and absorbed in the basement membrane. Anti-CNTN1 antibody (1:300) was shown to be positively correlated with the nodes of Ranvier in blood samples; all other antibodies (anti-NF155, anti-NF186, anti-CNTN2, and anti-CASPR1) were found to be negatively correlated.	After receiving immunoglobulin (IVIG, 0.4 g/kg) and methylprednisolone (1 g/day) as pulse treatment for five days, the patient's limb weakness improved. Given the concurrent presence of MN, rituximab (650 mg) was administered as a single dosage. Overall, the condition of the patient improved well and could walk independently.	None	Detected
Tang Y et al. [12]	2023	7 cases Mean age of onset 44.7 (ranging from 14 to 67 years) 4 (males) and 3 (females)	Before the infection became apparent, three patients experienced prodromic symptoms such as fever, cough, sore throat, and dermatitis of the right lower leg. The other patients had a progressive course and chronic onset, whereas only one patient had an acute-onset type with a period of remission and relapse. Regarding the sequence in which the two conditions expressed themselves, four patients had CIDP before nephropathy, two had CIDP and nephropathy commence at the same time, and one patient had nephropathy first. The early symptoms in every patient were limb weakness and/or numbness. Three patients experienced initial symptoms limited to their lower limbs. Three patients had more severe proximal limb weakness. Muscular atrophy in both hands affected only one case.	Investigations in 6 patients revealed nephrotic syndrome. An electrophysiological test revealed demyelination in each of the instances. All patients' nerve biopsies revealed mild to moderate mixed neuropathies, including axonal and demyelinating changes. All six patients had renal biopsies that revealed MN.	All the patients received corticosteroid therapy. Among them, 4 patients were given oral prednisone at doses of 50, 40, 80 and 5 mg/day respectively for one patient among these cyclophosphamide 50 mg twice daily was added, while patient 2 patients were initially treated with intravenous methylprednisolone at doses of 20 and 40 mg/day, followed by oral prednisone. One patient was given oral methylprednisolone 48 mg/day while another patient was also treated with cyclosporine A at doses of 50 mg twice a day. In addition, another 1 patient was treated with plasma exchange for five courses and rituximab at a dose of 1 g. Moreover, intravenous immunoglobulin was used in 1 patient, while hydroxychloroquine sulphate and cyclophosphamide were added for the patient who additionally suffered from SLE. Another 1 patient received seven rounds of plasma exchange therapy followed by 50 mg of cyclophosphamide orally every day. All patients received neurotrophic and diuretic medications. Following therapy, condition of each patient improved	None	Detected in four patients
Nazarali et al. [8]	2020	43 (male)	Aggressive generalized polyneuropathy	Increased levels of CSF (4.0 g/L) were observed and anti-CNTN1 IgG4 antibodies were detected. However, 12 months later patient presented with nephrotic syndrome. Renal biopsy was suggestive of stage 2 MN; additionally the biopsy revealed excessive CNTN1 staining within glomerulus in comparison to controls.	Intravenous immunoglobulin and tapering prednisone were started as a treatment plan, and the clinical and electrophysiologic responses were favourable while after the development of nephrotic syndrome, along with prednisone, cyclosporin was added, and protein levels dropped to 0.6 g/day in less than 18 months.	None	Detected

According to the findings, prednisolone is used quite a bit in the beginning and showed improvement for some studies but didn’t work for others. Each study had its own treatment methods. The most effective treatment for these combined conditions (MN and CIDP) is pulse methylprednisolone + rituximab (one to two doses) ± plasma exchange, based on consistent positive outcomes in both neurological and renal aspects.

The response patterns indicate that the best overall outcomes are achieved with rituximab following initial failure of steroids or IVIG. Partial or slower responses are observed with steroids alone or in combination with IVIG. Poor or unstable outcomes are associated with cyclosporine or tacrolimus without rituximab. For proteinuria control, the addition of cyclophosphamide to steroids has proven effective. These differences suggest that combined immunosuppressive strategies, particularly those including rituximab, are more effective than monotherapy, especially in patients with both neurological and renal involvement.

Hashimoto et al., while reviewing the literature, described that the average age at the development of CIPD was 47.6 ± 21.6 years (range: 9-81 years old; 36% >60 years old). The ratio of men to women was 10:4 (2.5:1). Nine instances had chronic onset of CIDP, four had acute onset (one month or less), and one had subacute onset (six weeks). In terms of immunotherapies' effectiveness on CIDP, corticosteroids were successful in 71.4% of patients when used in monotherapy. In all cases, plasma exchange combined with monotherapy was successful. When used in solitude, intravenous immunoglobulins proved beneficial in all patients. Four cases used combined immunotherapies, of which one resulted in success. Based on these findings, the majority of patients (79%) reacted well to immunotherapies like corticosteroids, plasma exchange, and IVIG at first; nevertheless, three cases (21%) were resistant to even combinations of treatments [7]. Similarly, Xu et al. reported in their analysis of the literature that early single-agent or combination immunotherapy elicited good responses, but in the final stages, eight of the 22 patients with concurrent MN and CIDP experienced distinct motor sequelae. Anti-CNTN1 antibody-associated autoimmune nodopathies with MN were observed in five cases. Of these patients, 40% had acute to subacute onset, the mean age at onset was late (60.2 ± 15.7 years, range 43-78 years), and males accounted for the majority of cases (male:female=4:1). Clinical signs included higher levels of protein in the CSF fluid, sensory-motor neuropathy, and sensory ataxia spurred on by proprioceptive dysfunction [6].

It has been elucidated that approximately 70% of individuals afflicted with idiopathic membranous glomerular nephropathies possess serum IgG4 autoantibodies directed against the glomerular podocyte antigen PLA2R. As a result, the inclusion of PLA2R antibody testing has brought about a significant transformation in the management of these nephropathies, potentially obviating the necessity for diagnostic biopsies in a considerable number of cases. Similarly, for patients who test negative for anti-PLA2R antibodies, the early detection of CNTN1 antibodies may offer a comparable advantage by potentially circumventing the need for further renal assessments and enabling an earlier initiation of treatment. Decreasing anti-CNTN1 titers also corresponded with improvements in clinical outcomes, indicating their usefulness as a prognostic indicator. Lower titers of PLA2R antibodies have been reported to occur before the commencement of remission, and high titers suggest a worse prognosis. Authors further highlighted their findings that similar to anti-PLA2R membranous glomerular nephropathies, serum CNTN1 antibody detection frequently occurred before the development of proteinuria, indicating their diagnostic utility. However, if antibody-mediated nephrotic syndrome is suspected in seronegative patients, renal biopsy continues to be considered [19].

This case differs from other ones in certain aspects. For example, in this case, MN developed 18 months after the initial diagnosis of CIDP. Previous reports have documented varying timelines: some patients developed MN prior to CIDP, others concurrently, and some after CIDP onset. This variability in disease progression contrasts with the delayed onset observed here [12]. Also the patient tested negative for PLA2R antibodies but positive for CNTN1 antibodies. This serological profile aligns with other rare reports where anti-CNTN1 antibodies are present in patients with CIDP and MN, suggesting a distinct antibody-mediated mechanism separate from typical PLA2R-associated MN [6]. The patient responded well to cyclosporine and prednisone therapy, with no immediate complications, but experienced a partial relapse during remission on cyclosporine. Some prior reports have described more refractory or relapsing courses requiring rituximab or other immunosuppressants. For example, patients with CIDP and anti-CNTN1 antibodies who were resistant to intravenous immunoglobulin and corticosteroids showed marked improvement after rituximab treatment [20]. The case describes a typical CIDP phenotype with proximal and distal weakness and sensory deficits, similar to other anti-CNTN1-positive cases. However, some reports have noted more severe or rapidly progressive neuropathy in anti-CNTN1-positive patients. A study highlighted that patients with anti-CNTN1 antibodies often present with aggressive neuropathy and poor response to standard therapies [16]. This case exhibits a delayed onset of MN after CIDP, a favorable initial response to standard immunosuppression, and a less aggressive neurological course compared to some prior reports.

Early detection of anti-CNTN1 antibodies has prognostic value in CIDP-MN overlap cases. It helps predict disease course, informs therapeutic strategy, and supports the idea of shared autoimmunity between the nervous system and kidneys. However, further prospective studies are needed to validate its role in clinical decision-making.

Strengths and limitations

The presentation of our case makes a meaningful contribution to the existing literature by supporting the potential role of anti-CNTN1 antibodies in the co-occurrence of CIDP and MN. This association aligns with emerging evidence suggesting a shared autoimmune mechanism. However, due to limited resources, we were unable to test for anti-THSD7A antibodies. As a result, we cannot rule out their involvement in the condition, which represents a limitation of our findings. Another limitation is the unavailability of additional patient information, which could not be retrieved from existing records.

## Conclusions

Based on our case analysis and comprehensive review of existing literature, the co-occurrence of MN and CIDP in anti-CNTN1-positive patients suggests a shared immunopathogenesis, which may require integrated treatment of both conditions. These findings highlight the need for personalized immunotherapy based on serological profiles and highlight the potential value of early anti-CNTN1 antibody detection as a prognostic and therapeutic marker in CIDP-MN cases. Future research should include large-scale, multicenter studies to determine the prevalence and clinical significance of anti-CNTN1 antibodies in patients with CIDP and MN. Additionally, further investigations should explore the underlying mechanisms through which anti-CNTN1 antibodies contribute to both peripheral nerve and kidney involvement.
